# Multiple Brain Abscesses Associated With Severe Bronchiectasis and Respiratory Infection: A Case Report

**DOI:** 10.7759/cureus.108956

**Published:** 2026-05-16

**Authors:** Sara Haddouga, Mohammed Sidayne, Jamal Ouachaou, Driouich Aicha, Fatimazahrae El Khettab, Youssef Zarrouki

**Affiliations:** 1 Anesthesiology and Intensive Care, Mohammed VI University Hospital of Marrakech, Faculty of Medicine and Pharmacy, Cadi Ayyad University, Marrakech, MAR

**Keywords:** arsd, brain abscess, bronchiectasis, septic shock, streptococcus pneumonia

## Abstract

Brain abscess is a rare but life-threatening intracranial infection most commonly resulting from hematogenous dissemination or contiguous spread. Pulmonary infections, including bronchiectasis, may represent a potential source of systemic infection.

We report the case of a 50-year-old woman with type 2 diabetes mellitus admitted for acute hypoxemic respiratory failure secondary to severe bronchiectasis-associated infection complicated by acute respiratory distress syndrome (ARDS). Respiratory samples identified *Streptococcus pneumoniae* and Influenza A virus. Blood cultures remained negative.

The patient initially improved under empirical antibiotic therapy but subsequently developed neurological deterioration on hospital day 5, characterized by seizures and nystagmus. Brain magnetic resonance imaging (MRI) revealed multiple bilateral cerebral abscesses with ring enhancement and diffusion restriction consistent with pyogenic abscesses. Transthoracic echocardiography showed no evidence of infective endocarditis, although transesophageal echocardiography was not performed.

Despite escalation of antimicrobial therapy and intensive care management, the patient developed refractory septic shock and multiorgan failure, resulting in death.

## Introduction

Brain abscess is a severe intracranial infection associated with significant morbidity and mortality. It may result from direct extension of infection or hematogenous spread from distant infectious foci [1].

Bronchiectasis is a chronic pulmonary disease characterized by irreversible bronchial dilation and recurrent infections [2,3]. In severe cases, systemic dissemination may occur, particularly in patients with comorbidities such as diabetes mellitus.

Although pulmonary infections are recognized causes of brain abscess, this complication remains rare in bronchiectasis [1,4]. Infective endocarditis remains the most common source of hematogenous cerebral dissemination and must be systematically excluded [2].

We report a rare case of multiple brain abscesses occurring in a patient with severe bronchiectasis-associated infection and acute respiratory distress syndrome (ARDS).

## Case presentation

A 50-year-old woman with type 2 diabetes mellitus presented with a four-day history of fever and productive cough with purulent sputum. On admission, she was febrile, tachycardic, and in severe respiratory distress, requiring immediate transfer to the intensive care unit.

**Figure 1:**
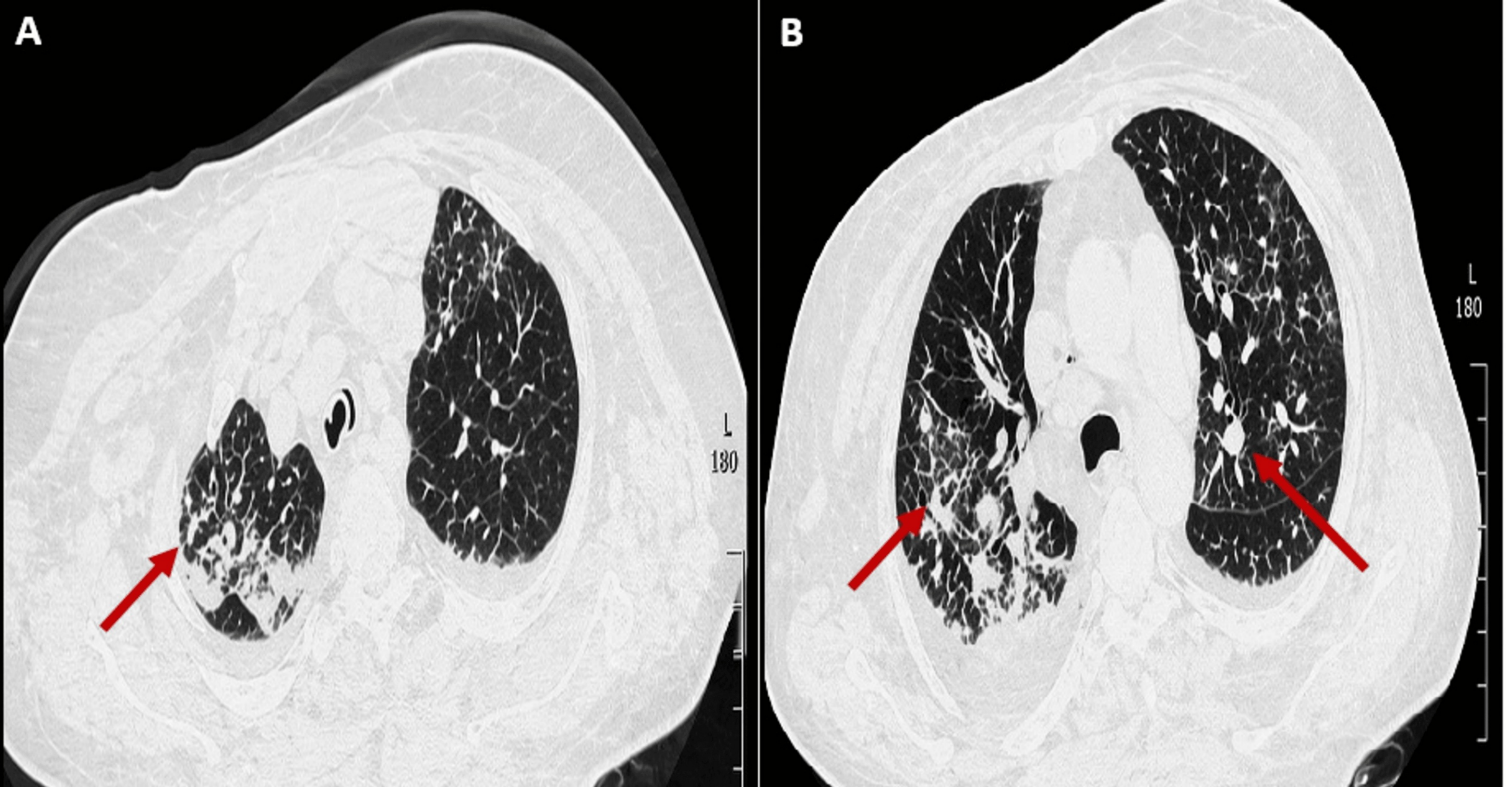
Axial thoracic CT scans (A and B) showing bronchiectasis, indicated by arrows

Chest computed tomography (CT) revealed cylindrical bronchiectasis with a characteristic tram-track appearance (Figure 1).

Initial laboratory investigations demonstrated marked leukocytosis (19,960/mm³, neutrophils 90%) and elevated C-reactive protein (160 mg/dL). Renal function and electrolytes were within normal limits.

Respiratory microbiological samples identified *Streptococcus pneumoniae* and Influenza A virus. Blood cultures remained negative. The patient required intubation and mechanical ventilation. She subsequently developed ARDS (PaO₂/FiO₂ <200 mmHg). Empirical antibiotic therapy with ceftriaxone and amikacin was initiated.

During the ICU course, she developed septic shock requiring norepinephrine infusion, with lactate monitoring for tissue hypoperfusion. Despite initial stabilization, neurological deterioration occurred on hospital day 5, characterized by generalized seizures, nystagmus, and altered mental status. Seizures were managed with anticonvulsants.

**Figure 2:**
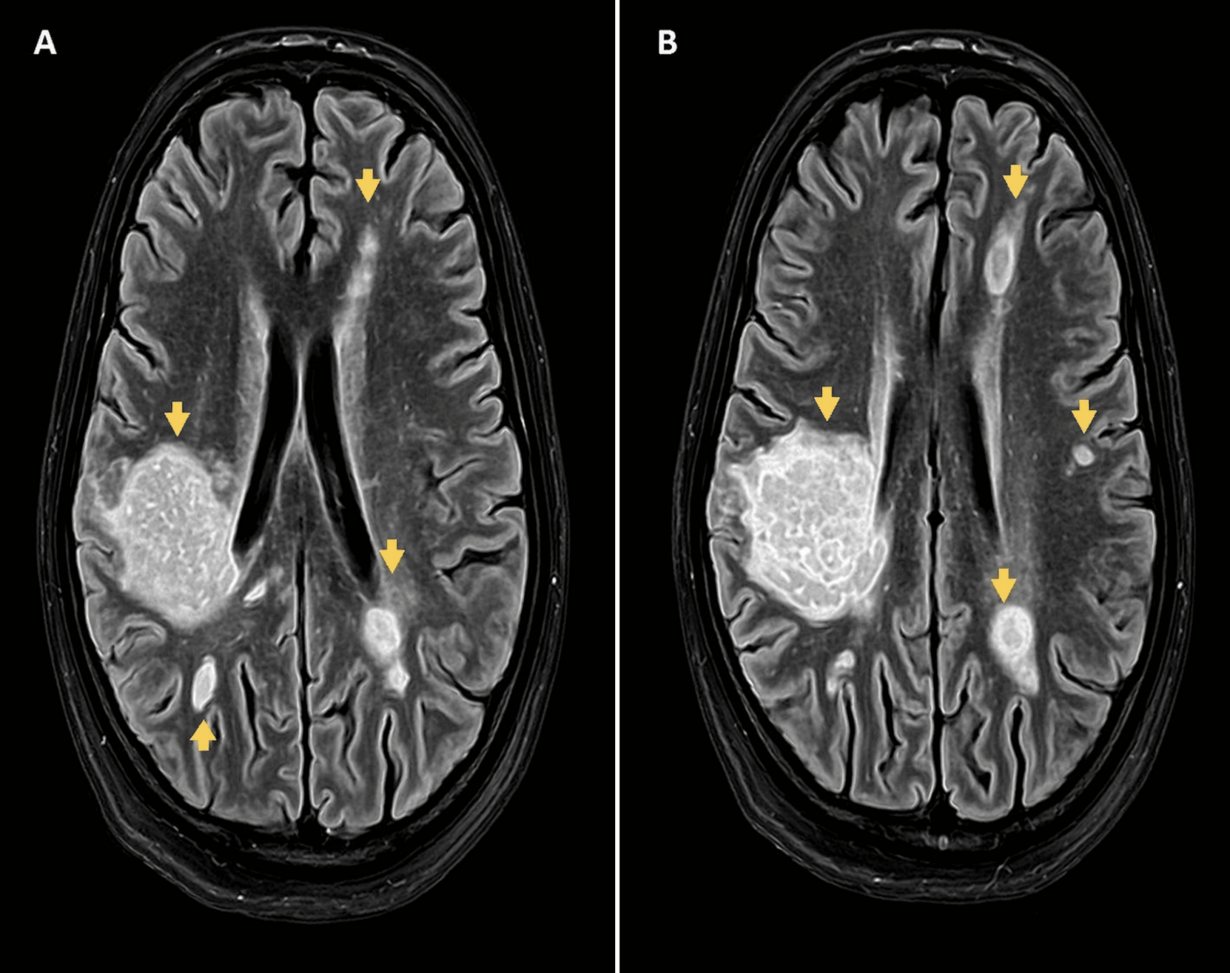
Axial brain MRI images (A and B) demonstrating cerebral abscesses (arrows) MRI, magnetic resonance imaging.

Brain magnetic resonance imaging (MRI) demonstrated multiple multiloculated cerebral abscesses involving cortico-subcortical regions (Figure 2).

Findings included ring enhancement, diffusion restriction, vasogenic edema, and mild mass effect, consistent with pyogenic abscesses [1,5].

**Figure 3:**
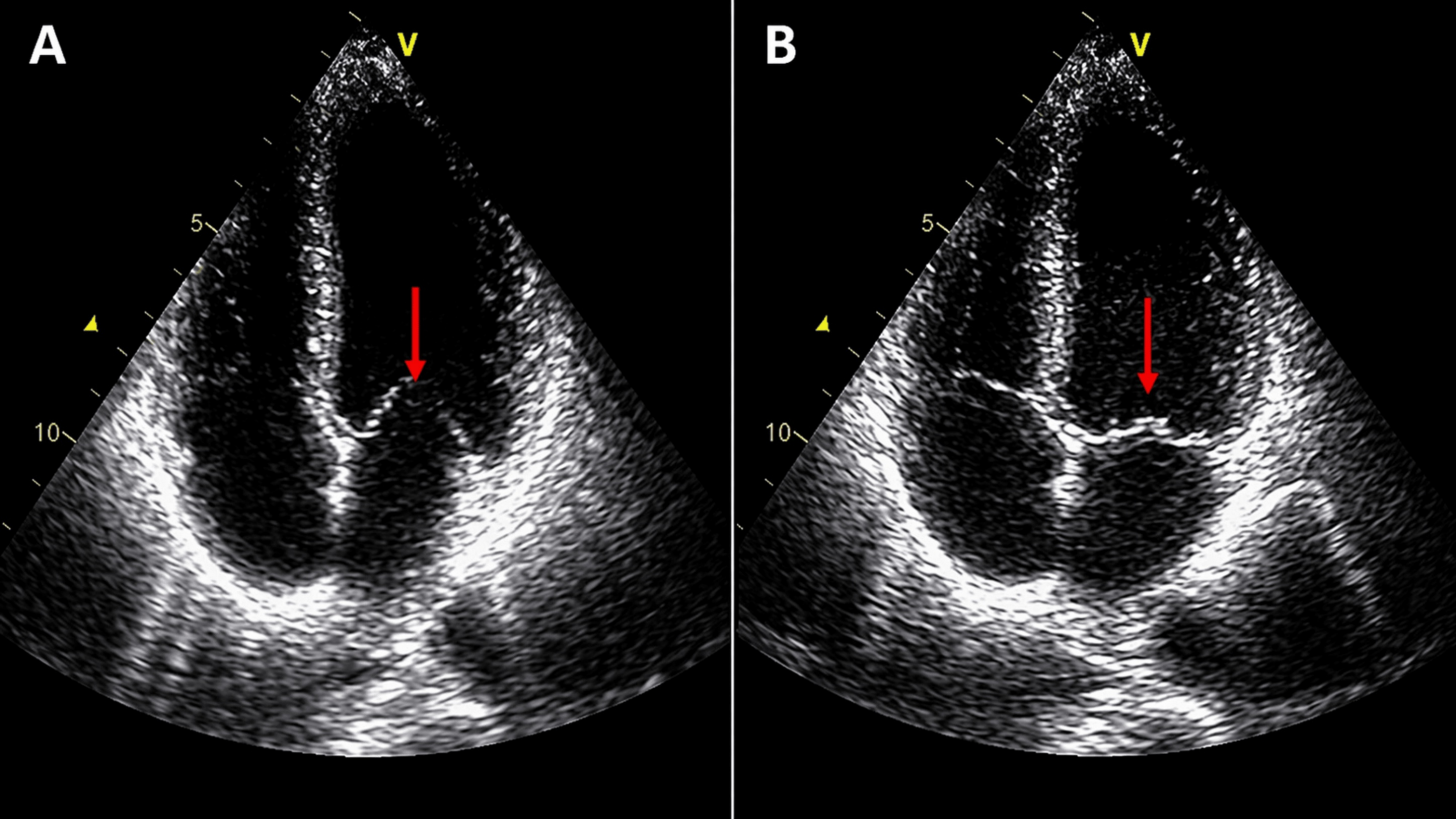
Echocardiographic images (A and B) obtained in systole and diastole, showing normal valve motion without vegetations (arrows)

**Figure 4:**
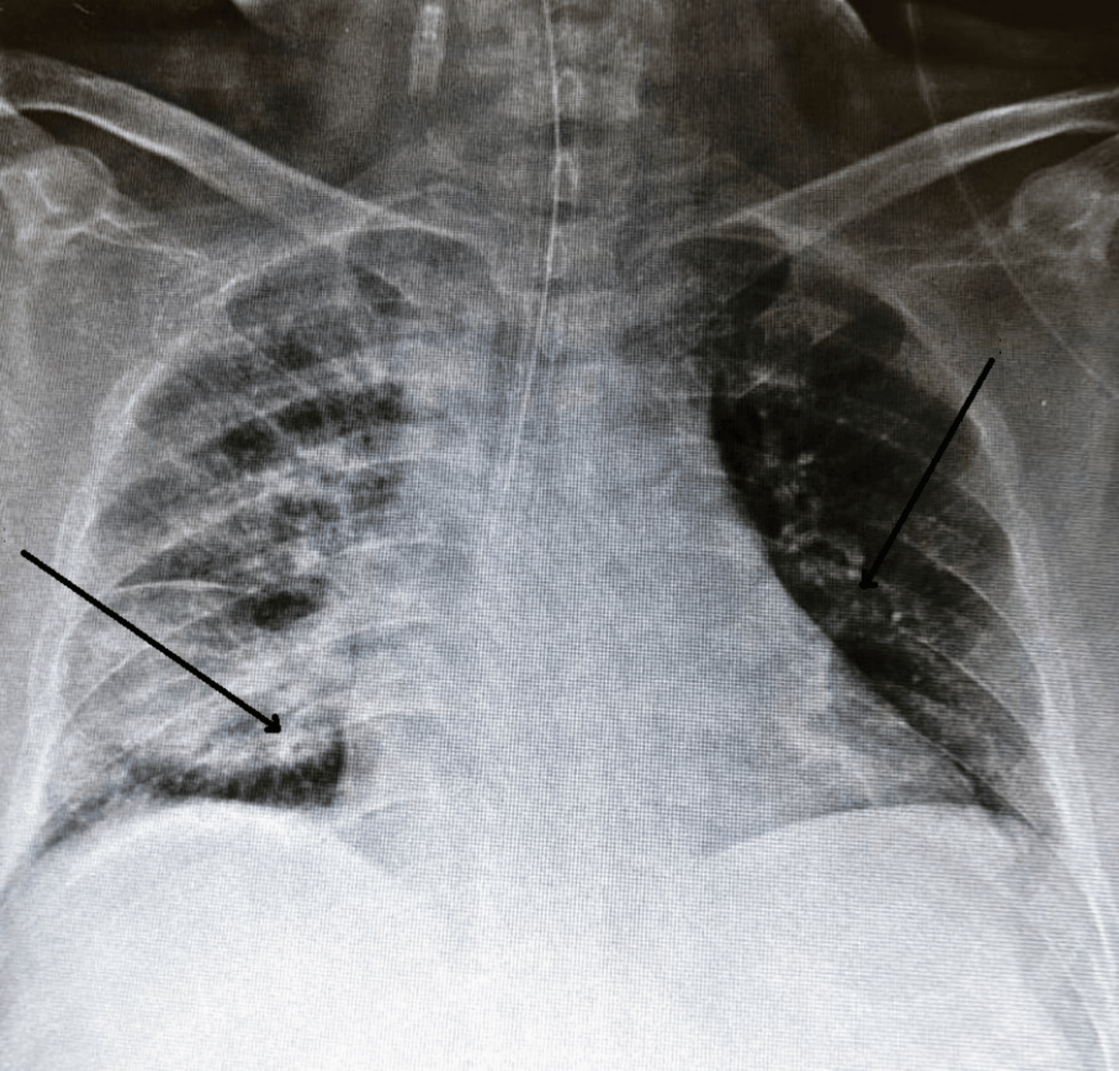
Chest X-ray image with known bronchiectasis

HIV serology was negative. Transthoracic echocardiography showed no evidence of infective endocarditis (Figure 3), although transesophageal echocardiography was not performed, representing a diagnostic limitation. Chest X-ray showed known bronchiectasis without new abnormalities (Figure 4).

Antibiotic therapy was escalated to meropenem, vancomycin, and metronidazole. Neurosurgical intervention was not performed due to multiplicity and deep lesion location.

Despite maximal intensive care support, the patient progressed to refractory septic shock and multiorgan failure and died three days after neurological deterioration.

## Discussion

Brain abscess is a rare but severe intracranial infection associated with significant morbidity and mortality despite advances in imaging and antimicrobial therapy [1]. Hematogenous dissemination is a recognized mechanism and typically results in multiple lesions involving both cerebral hemispheres [1,2]. In our patient, the MRI pattern of multiple cortico-subcortical abscesses strongly suggested hematogenous spread.

Bronchiectasis is characterized by chronic airway inflammation, impaired mucociliary clearance, and recurrent pulmonary infections, which may facilitate systemic bacterial dissemination during severe exacerbations [3]. However, brain abscess remains an uncommon complication of bronchiectasis, with only a few cases reported in the literature [4,5].

Several factors may have contributed to the severity of infection in this case, including diabetes mellitus and coexisting Influenza A virus infection with *S. pneumoniae*. Viral-bacterial coinfection is known to increase the risk of invasive bacterial disease and severe pulmonary infection [6,7].

Although *S. pneumoniae* was identified in respiratory samples, blood cultures remained negative, and no microbiological confirmation from the cerebral lesions was available. Therefore, the pulmonary origin of the brain abscesses remains strongly suspected but cannot be definitively proven. Nevertheless, the temporal association between severe respiratory infection and subsequent neurological deterioration, together with the radiological pattern of multiple abscesses, supports probable hematogenous dissemination.

Alternative infectious sources were investigated. Transthoracic echocardiography showed no evidence of infective endocarditis; however, transesophageal echocardiography was not performed, which limits the ability to fully exclude a cardiac source. No otogenic, sinus, abdominal, genitourinary, or skin source of infection was identified clinically.

Neurological deterioration occurred on hospital day 5 after initial respiratory stabilization, emphasizing the importance of close neurological monitoring in critically ill patients with severe pulmonary infection. New neurological symptoms such as seizures, altered mental status, and focal deficits should prompt urgent neuroimaging to exclude intracranial complications.

Management of brain abscess relies on prompt administration of antibiotics with adequate central nervous system penetration and neurosurgical drainage when feasible [1,2]. In our patient, antimicrobial therapy was escalated to meropenem, vancomycin, and metronidazole. Surgical drainage was not performed because of the multiplicity and deep location of the lesions.

Despite aggressive intensive care management, the patient developed refractory septic shock and multiorgan failure. This case highlights the potential severity of bronchiectasis-associated infection complicated by suspected hematogenous cerebral dissemination and underlines the importance of early recognition of neurological complications in critically ill patients.

## Conclusions

This case highlights that severe bronchiectasis-associated respiratory infection may rarely be complicated by suspected hematogenous dissemination, leading to multiple brain abscesses. Although a definitive microbiological link could not be established, the temporal association between severe pulmonary infection and subsequent neurological deterioration, together with the radiological pattern of multiple lesions, strongly supports this hypothesis.

The development of new neurological symptoms during ICU hospitalization, particularly seizures or altered mental status, should prompt urgent neuroimaging in critically ill patients with severe pulmonary infection. Early recognition and multidisciplinary management are essential, as prognosis remains poor in patients with multiple abscesses and septic shock.
